# MiRNA-206 suppresses PGE2-induced colorectal cancer cell proliferation, migration, and invasion by targetting TM4SF1

**DOI:** 10.1042/BSR20180664

**Published:** 2018-09-19

**Authors:** Young Ran Park, Seung Young Seo, Se Lim Kim, Shi Mao Zhu, Sungkun Chun, Jung-Mi Oh, Min Ro Lee, Seong Hun Kim, In Hee Kim, Seung Ok Lee, Soo Teik Lee, Sang Wook Kim

**Affiliations:** 1 Department of Internal Medicine, Chonbuk National University Hospital, Chonbuk National University Medical School, Jeonju, Republic of Korea; 2 Research Institute of Clinical Medicine of Chonbuk National University-Biomedical Research Institute, Chonbuk National University Hospital, Chonbuk National University Medical School, Jeonju, Republic of Korea; 3 Department of Physiology, Medical School, Chonbuk National University, Jeonju, Republic of Korea; 4 Department of Surgery, Chonbuk National University Hospital, Chonbuk National University Medical School, Jeonju, Republic of Korea

**Keywords:** colorectal cancer, invasion, miR-206, migration, TM4SF1

## Abstract

MiRNA (miR)-206 plays a tumor suppressor role in various cancer types. Here, we investigated whether miR-206 is involved in prostaglandin E2 (PGE2)-induced epithelial–mesenchymal transition (EMT) in colorectal cancer (CRC) cells through the targetting of transmembrane 4 L six family member 1 (TM4SF1).

The effect of PGE2 on growth and apoptosis of CRC cells was evaluated using the MTT assay and flow cytometry analysis, respectively. TM4SF1 and miR-206 expression levels were determined with quantitative polymerase chain reaction (qRT-PCR) in CRC tissues and cell lines. The concentration of PGE2 in the serum of CRC patients and healthy controls was measured with an ELISA kit. A miR-206 or TM4SF1 construct was transfected into cells with PGE2. Transwell migration and invasion assays were used to examine cell migration and invasion properties. Additionally, a luciferase assay was performed to determine whether TM4SF1 was directly targetted by miR-206.

We found that miR-206 was down-regulated and TM4SF1 was up-regulated in human CRC tissues and cell lines. Moreover, miR-206 was negatively correlated with TM4SF1 expression. Bioinformatics analysis and a luciferase reporter assay revealed that miR-206 directly targetted the 3′-untranslated region (UTR) of TM4SF1, and TM4SF1 expression was reduced by miR-206 overexpression at both the mRNA and protein levels. Additionally, PGE2 significantly suppressed the expression of miR-206 and increased the expression of TM4SF1 in CRC cells. PGE2 induction led to enhanced CRC cell proliferation, migration, and invasion. Moreover, the overexpression of miR-206 decreased CRC cell proliferation, migration, and invasion compared with control group in PGE2-induced cells, and these effects could be recovered by the overexpression of TM4SF1. Overexpression of miR-206 also suppressed the expression of β-catenin, VEGF, MMP-9, Snail, and Vimentin and enhanced E-cadherin expression in PGE2-induced cells. These results could be reversed by the overexpression of TM4SF1. At last, up-regulation of miR-206 suppressed expression of *p*-AKT and *p*-ERK by targetting TM4SF1 in PGE2-induced cells.

Our results provide further evidence that miR-206 has a protective effect on PGE2-induced colon carcinogenesis.

## Introduction

Colorectal cancer (CRC) is one of the most common malignant tumors and one of the major causes of cancer-related deaths, with most CRC patients dying due to metastasis [1–3]. Multiple complex mechanisms regulate cancer metastasis, including controlled epithelial–mesenchymal transition (EMT), increased cancer stemness, and angiogenesis. Amongst them, EMT is identified as one of the central steps in cancer metastasis, which is characterized by an increased in EMT transcription factors (EMT-TFs), such as β-catenin, VEGF, MMP-9, Snail, and Vimentin and decreased expression of E-cadherin [4]. In the present study, we investigated the effect of miR-206 on the regulation of PGE-induced EMT in CRC cells, and further evaluated the underlying mechanism.

Recently, it has been reported that cyclooxygenase 2 (COX-2) and prostaglandin E2 (PGE2) regulate cancer progression and metastasis [5,6]. Overexpression of COX-2 in tumor-associated macrophages promotes pro-metastatic effects of breast cancer cells by regulating MMP-9 and EMT and Akt pathway, and the COX-2/PGE2 axis mediates cell invasion in EGF-induced ovarian cancer [7,8]. Especially, PGE2 produced by inducible COX-2 and mPGES-1 promotes cancer cell proliferation *in vitro* and *in vivo*, and induced EMT in prostate cancer [9,10]. Moreover, many molecular pathways have been identified as downstream of PGE2 to promote cell migration and invasion, including the PI3K/Akt, β-catenin/TCF-4, and JAK2/STAT3 signaling pathways [11–14]. These results have shown that PGE2 may act as an important factor in the development, migration, and invasion of CRC cells. However, its essential role in regulating the migration and invasion of CRC cells remains unclear.

MiRNAs are a class of regulatory non-coding RNAs that bind to a target site in the 3′-untranslated region (UTR) of target mRNAs [15]. MiRNAs have been involved in several cellular processes, including survival, proliferation, apoptosis, and EMT in many types of cancers [16]. Amongst these miRNAs, miR-206 has been identified as a tumor suppressor in many human malignancies, including breast, rhabdomyosarcoma, renal, lung, laryngeal, endometriosis, colorectal, and gastric cancers [17–24]. Several pathways or molecules including TGF-β, PI3K/Akt/mTOR, and VEGF have been identified as downstream mediators of miR-206 in various cancers. In laryngeal cancer, down-regulation of miR-206 increased cell proliferation, migration, and invasion by regulating VEGF expression [21]. Moreover, the overexpression of miR-206 suppressed EMT through targetting TGF-β signaling in estrogen receptor positive breast cancer cells [25]. In non-small cell lung cancer, miR-206 decreased hepatocyte growth factor (HGF)-induced cell EMT and angiogenesis by targetting c-MET/PI3K/AKT/mTOR pathway [26]. Recently, Liu et al. [27] reported that miR-206 inhibits cell progression in HNSCC cancer through up-regulating histone deacetylases 6 (HDAC6) via PTEN/AKT/mTOR pathway. In CRC, miR-206 suppressed cell proliferation and migration through the down-regulation of NOTCH3, and low expression of miR-206 significantly increased cell proliferation via activation of Kruppel-like factor 4 (KLF4) [28,29]. Moreover, miR-206 expression was significantly lower in CRC tissues than adjacent normal tissues [30]. Ren et al. [31] showed that miR-206 expression markedly suppressed cell invasion and induced apoptosis by directly targetting FMNL2 in CRC. However, the role of miR-206 in PGE2-induced EMT in CRC remains unknown.

We used bioinformatics tools, TargetScan, PicTar, and MicroRanda to identify miRs that may bind to the 3′- UTR of Transmembrane 4 L Six Family Member 1 (TM4SF1). Five candidate miRs, including miR-9, miR-30a, miR-206, miR-107, and miR-181d, were predicted to target TM4SF1. In our preliminary study, we found that miR-9, miR-30a, and miR-206 could each inhibit the migration or invasion of CRC cells, but miR-107 and miR-181d could not. Subsequently, we have reported that miR-9 and miR-30a inhibit CRC migration and invasion by targetting TM4SF1 [32,33]. Now, we seek to investigate whether miR-206 can inhibit CRC metastasis by inhibiting TM4SF1.

TM4SF1 is a member of the tetraspanin L6 domain family [34], which includes TM4SF1/L6, TM4SF4/ILTMP, and TM4SF5/L6H. Amongst these families, TM4SF1 was initially identified as tumor-associated antigen L6, and the gene mapped to chromosomal region 3q21-3q25 [35–37]. TM4SF1 is increased in epithelial cancer cells and regulates cell motility and invasion in colorectal and liver cancers [32,38]. Kleivei et al. [39] suggested that TM4SF1 expression was up-regulated in primary colorectal, liver metastasis, and carcinomatosis by using gene expression profiles, and TM4SF1 promoted breast cancer cell migration and invasion, and apoptosis via PI3K/AKT/mTOR pathway [30]. Similar results were obtained with HCT 116 cells (Supplementary Figure S1). In our previous study, TM4SF1 was also up-regulated in CRC patients, and regulated metastatic potential of CRC cells via the activation of EMT regulators, MMP-2/9, VEGF, and β-catenin [32,33]. These reports have shown that TM4SF1 increases tumor migration and invasion, suggesting that it may act as an important regulator in the development and metastases of CRC.

**Figure 1:**
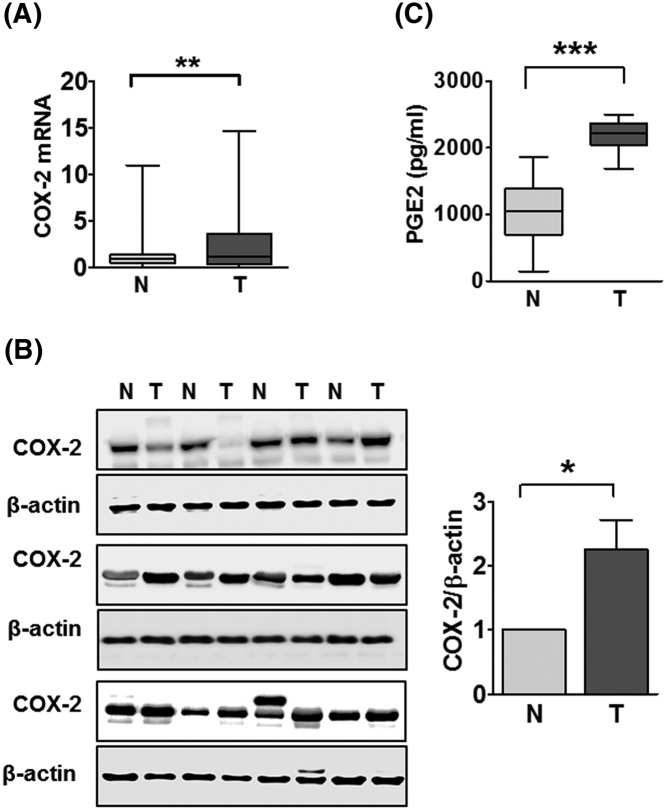
PGE2 concentration and COX-2 expression (**A**) The qRT-PCR for COX-2 expression in 60 CRC tissues and paired adjacent normal tissues. (**B**) Western blot analysis for COX-2 expression in four CRC patients and paired normal tissues. (**C**) Concentration of PGE2 in human serum. An ELISA assay was used to measure 60 CRC serum samples and 30 human normal serum samples. *P<0.05, **P<0.01, and ***P<0.001 compared with normal.

In the present study, we investigated the role of miR-206 and TM4SF1 in PGE2-induced CRC metastasis *in vitro*. We found that overexpression of miR-206 had a protective effect on PGE2-induced proliferation, migration, and invasion through the inhibition of TM4SF1. Moreover, transfection of miR-206 suppressed expression of β-catenin, VEGF, MMP-9, Snail, and Vimentin, and increased the expression of E-cadherin by targetting TM4SF1 in PGE2-induced CRC cells. The overexpression of miR-206 also inhibited the expression of *p*-AKT and *p*-ERK by inhibiting TM4SF1 in PGE2-induced cells.

Thus, miR-206 shows a critical role in the pathogenesis and development of CRC and suggests a possible direction in the treatment of PGE2-induced CRC.

## Materials and methods

### CRC clinical samples and cell cultures

Sixty CRC tissues and adjacent normal tissues were freshly obtained from the Biobank of Chonbuk National University Hospital, frozen in liquid nitrogen, and stored at −80^°^C until further study. The demographic, clinical, and pathological characteristics were obtained from the medical records and are shown in Table 1. The study design was approved by the institutional review board of Chonbuk National University Hospital (IRB number; 2016-04-018). Human CRC cell lines (HCT116, Caco-2, SW480, DLD-1, HT29, and LOVO) were purchased from the American Type Culture Collection (ATCC, Manassas, VA, U.S.A.). All cell lines were maintained using Roswell Park Memorial Institute (RPMI) 1640 medium supplemented with 10% FBS and 1% penicillin/streptomycin at 37°C in a humidified atmosphere containing 5% CO_2_. The human colonic fibroblast cell line, CCD-18co, was obtained from the Korean Cell Line Bank (KCLB, Seoul, Korea) and cultured in Dulbecco Modified Eagle’s Media (DMEM) supplemented with 10% FBS. All cell culture reagents were obtained from Gibco (Carlsbad, CA, U.S.A.).

**Table 1:** Clinic pathological characteristics in 60 of CRCs

Variables	Number, *n* (%)
Gender	
Male	36 (60.0)
Female	24 (40.0)
Age (years)	
<65	30 (50.0)
≥65	30 (50.0)
Histological differentiation	
Well	9 (15.0)
Moderate	44 (73.3)
Poor	7 (11.6)
Tumor status (T)	
T1–T2	20 (33.3)
T3–T4	40 (66.6)
Lymph node metastasis (N)	
No	35 (58.3)
Yes	25 (41.6)
AJCC	
I+II	33 (55.0)
III+IV	27 (45.0)

Abbreviation: AJCC, American Joint Committee on Cancer.

### Cell transfection and treatments with PGE2

miR-206 and the miR-negative control (NC) were purchased from GenePhama (Shanghai, China). For transient transfections, the cells were cultured to 70–90% confluence and transfected with 50 nM of each respective miR or a NC with Lipofectamine 2000 (Invitrogen, Carlsbad, CA, U.S.A.) according to the manufacturer’s instructions. After transfection, cells were harvested or incubated with PGE2 for the following experiments.

### RNA extraction and qRT-PCR analysis

Total RNA was extracted using Trizol reagent (Invitrogen) from either frozen tissues or cells according to the manufacturer’s protocols. For cDNA synthesis, reverse transcription was performed using 1 μg of total RNA with the Go Script Reverse Transcription System (Promega, Maddison, MA, U.S.A.). The TM4SF1 gene was detected by using the TOPreal™ qPCR 2X PreMIX containing SYBR Green (Enzynomics, Daejeon, Korea) using a QuantStudio™ Real-Time PCR system (Applied Biosystems, CA, U.S.A.). For qRT-PCR, the β-2 microglobulin (β2M) gene and RNU48 were used as endogenous controls for TM4SF1 mRNA and miR-206 expression analysis, respectively. The following primers were used: TM4SF1 forward, 5′-TCGCGGCTAATATTTTGCTT-3′, reverse, 5′-TGCAATTCCAATGAGAGCAG-3′; B2M forward, 5′-CCTGAATTGCTATGTGTCTGGG-3′, revers, 5′-TGATGCTGCTTACATGTCTCGA-3′. The 2^−ΔΔ*C*^_t_ method was used to calculate the fold change for miR-206 and TM4SF1 mRNA expression relative to the control.

### Protein extraction and Western blot analysis

Total protein was isolated from cells or tissues using protein cell lysis buffer (50 mM Tris pH 7.5, 150 mM NaCl, 1 mM EDTA, 1% NP-40, 0.5% sodium deoxycholate, and 1% Protease Inhibitor Cocktail (1 mM PMSF, 1 mM sodium ortho-vanadate, and 20 mM sodium fluoride). Lysates were extracted by centrifugation at 13000 rpm for 20 min at 4°C. Equal amounts of whole-cell lysates were analyzed by SDS-PAGE and transferred to PVDF membranes (Bio-Rad, CA, U.S.A.). Membranes were blocked with 5% skimmed milk and then immunoblotted at 4°C using the following primary antibodies: anti-COX-2 (1:1000, BD Biosciences, U.S.A.), anti-TM4SF1 (1:1000, Thermo Pierce, IL), anti-E-cadherin (1:1000, Cell Signaling Technology, U.S.A.), anti-β-catenin (1:1000, Cell Signaling Technology), anti-VEGF (1:500, Santa Cruz Biotechnology, U.S.A.), anti-MMP-9 (1:1000, Cell Signaling Technology), anti-*p*-AKT (1:1000, Cell Signaling Technology), anti-*p*-ERK (1:1000, Cell Signaling Technology), PCNA (1:1000, Cell Signaling Technology), and anti-β-actin (1:10000, Sigma-Aldrich, MO). Membranes were probed at room temperature for 1 h with a goat anti-rabbit or anti-mouse IgG conjugated to horseradish peroxidase (Santa Cruz Biotechnology). Protein expression was detected using the Dyne ECL STAR Western Blot Detection kit (Dyne Bio, Seoul, Korea) and a chemiluminescent image system (Fusion Solo system, Villber Lourmat). All experiments were performed in triplicate.

### Cell proliferation assay

Cell proliferation assays were based on the MTT method. HCT116 and Caco-2 cells (2 × 10^3^) were seeded in 96-well plates, transfected with 50 nM of miR-206 or TM4SF1 construct, and incubated for 24–72 h. The supernatants were removed and MTT solution (1 mg/ml) was added to each well and incubated at 37°C for 3 h. DMSO was added to each well and optical density (OD) at 450 nm was measured with a microplate reader. All experiments were performed in triplicate.

### Motility assay

A 24-well transwell plate (SPL, Gyeonggi-do, Korea) with an 8 μm pore size membrane was used to measure the migration and invasion ability of HCT116 and Caco-2 cells. For the invasion assay, the upper chambers were coated with diluted Matrigel (BD Biosciences, U.S.A., 1:10) at 37^°^C for 4–5 h. For the migration assay, 5 × 10^4^ of transfected cells suspended in serum-free medium were plated in the upper chamber. The chambers were inserted into the wells and incubated at 37^°^C for 48 h. The cells remaining on the upper surface of the membranes were removed, whereas the cells moving to the lower surface were fixed and stained with 0.1% crystal violet, and counted in five random fields.

### DNA construct and luciferase reporter assay

The 3′-UTR sequences of the TM4SF1 gene were cloned into the pmirGLO luciferase vector (Promega), which was named TM4SF1 3′-UTR-WT. Using TM4SF1 3′-UTR-WT as a template, point mutations in the putative miR-206-binding seed regions were amplified with a site-directed mutagenesis kit (Enzynomic, Daejeon, Korea), which was named TM4SF1 3′-UTR-MT. The primers for TM4SF1 3′-UTR-WT were: 5′-GCTCGCTAGCCTCGAGAATGAGGAAACAAACCACC-3′ (forward) and 5′-CGACTCTAGACTCGATGGGAAACATCATACAAGCA-3′ (reverse). The primers for TM4SF1 3′-UTR-MT were: 5′-GGATAAAAATAAATCACTATTGTATA-3′ (forward) and 5′-TATACAATAGTGATTTATTTTTATCC-3′ (reverse). A dual-luciferase reporter assay was performed to validate TM4SF1 as a direct target gene of miR-206. HCT116 and Caco-2 cells were maintained in 24-well plates for 12 h and co-transfected with 200 ng of TM4SF1 3′-UTR-WT (WT) or TM4SF1 3′-UTR-MT (MT), and 50 nM of miR-206 mimics or a NC for 48 h. Luciferase activity was measured using the Dual-Luciferase Reporter Assay system (Promega) following the manufacturer’s protocols. *Renilla* luciferase was used for normalization, and all experiments were performed independently in triplicate and repeated three times. A plasmid DNA containing the full ORF of the TM4Sf1 gene was generously donated by Dr R. Roffler (Academia Sinica, Taipei, Taiwan).

### Measurement of PGE2

Serum samples of CRC patients and normal serum were obtained from the Biobank of Chonbuk National University Hospital and Jeju National University Hospital, a member of the National Biobank of Korea. The concentrations of PGE2 in human serum were determined by a competitive ELISA kit (Enzo Life Science, U.S.A.) according to the manufacturer’s instruction. Absorbance was determined at 405 nm using a microplate reader.

### Cell apoptosis analysis

The Annexin-FITC Apoptosis Detection Kit (BD Biosciences, Franklin Lake, NJ, U.S.A.) was used to measure cell apoptosis. After transfection and treatment, cells were harvested and washed in PBS. Cells were added to 0.5 ml binding buffer and Annexin V-FITC and stained in the dark for 15 min at room temperature. The apoptotic cells were measured by a BD Accuri™ C6 flow cytometer (BD Biosciences). Cells positive for Annexin V-FITC staining were considered apoptotic cells.

### Statistical analysis

The data were calculated as the mean ± S.D. from at least three independent experiments. All quantitative data were calculated using the Student’s *t*-test, non-parametric test (Mann–Whitney U tests) and ANOVA when three or more groups were compared. The Pearson correlation analysis was used to evaluate the association between the expression of miR-206 and TM4SF1. *P* values <0.05 were considered statistically significant.

## Results

### COX-2 and PGE2 are highly expressed in CRC tissues and serum

We initially examined the expression of COX-2 mRNA in CRC specimens and the adjacent normal tissues by qRT-PCR. The expression of COX-2 was significantly up-regulated in CRC tissues as compared with paired normal tissues (Figure 1A). In addition, the protein expression of COX-2 was higher in CRC tissues (T) than in paired normal specimens (N) (Figure 1B). Next, we determined the concentration of PGE2 in normal and CRC patient serums by using an ELISA assay. Compared with normal serum, the concentration of PGE2 was significantly up-regulated in CRC serum (Figure 1C). These results were consistent with pro-inflammatory regulators such as COX-2 or PGE2, promoting tumor progression and metastasis in CRC [5].

### Correlation analysis of miR-206 and TM4SF1 expression in human CRC tissues and cell lines

**Figure 2:**
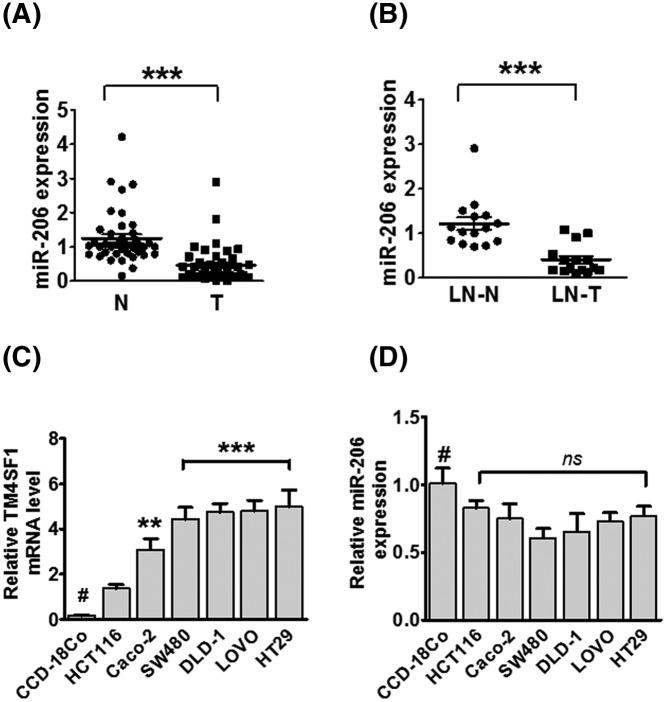
miR-206 is down-regulated in CRC tissues and in human CRC cell lines (**A**) Relative expression of miR-206 in CRC tissues (T) and paired normal tissues (N) was detected by qRT-PCR. (**B**) Relative expression of miR-206 in lymph node metastatic tissues (LN-T) and non-lymph node metastatic tissues (LN-N). Expression was normalized to RNU48. (**C**) TM4SF1 expression in HCT116, Caco-2, SW480, DLD-1, LOVO, and HT29 cells using qRT-PCR. (**D**) The relative expression of miR-206 in CRC cell lines as compared with the human colonic fibroblast cell line, CCD-18co (#); ^**^P<0.01, and ^***^P<0.001.

To determine miR-206 expression in CRC, we analyzed the expression level of miR-206 in CRC tissues and paired adjacent normal tissues. We found that miR-206 expression was significantly down-regulated in CRC patients as compared with the normal group (*P*<0.0001, Figure 2A). Moreover, miR-206 expression was lower in lymph node metastatic tissues (LN-T) than in non-lymph node metastatic tissues (LN-N) in paired lymph node metastatic CRC patients (Figure 2B). By statistical analysis, we found that miR-206 expression was negatively correlated with TM4SF1 mRNA expression and not significantly associated with TM4SF1 in human CRC tissues (not shown). We also analyzed the expression of miR-206 and TM4SF1 mRNA expression level in six CRC cell lines (HCT116, CaCO2, SW480, DLD-1, LOVO, and HT29). The human fibroblast cell line, CCD-18co, was used as normal control. As shown in Figure 2C, the expression of TM4SF1 was markedly up-regulated in CRC cell lines compared with expression in CCD-18co cells, while miR-206 expression was down-regulated in these cell lines, it was not significantly different from normal control (Figure 2D). These findings indicate that miR-206a is inversely associated with TM4SF1 expression in CRC tissues and cells.

### TM4SF1 is a direct target of miR-206

**Figure 3:**
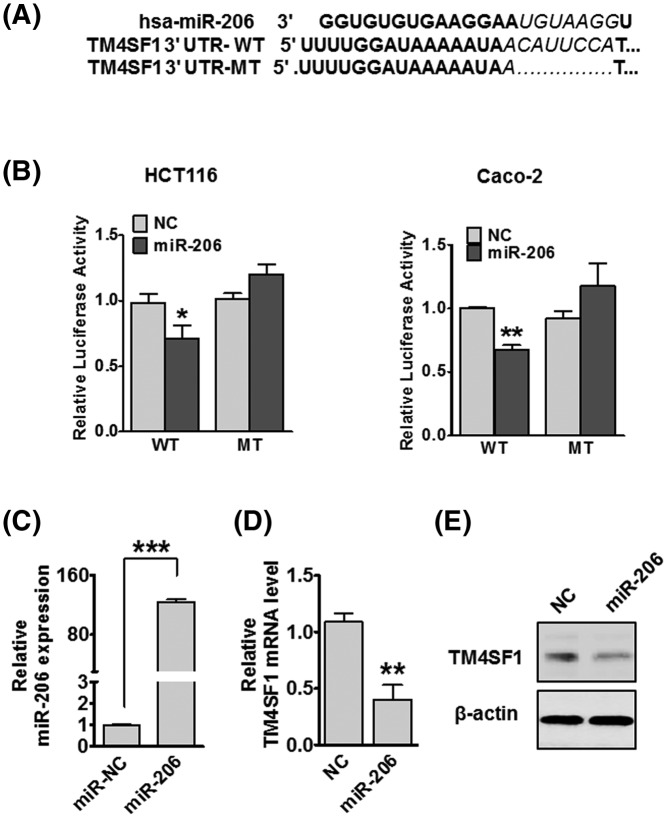
miR-206 directly targets TM4SF1 in CRC cells (**A**) Bioinformatics analysis predicted miR-206-binding sites of miR-206 in TM4SF1 3′-UTR. (**B**) Relative luciferase activity of wild-type or mutant TM4SF1 in HCT116 and Caco-2 cells. (**C**) Relative miR-206 expression in HCT116 cells following transfection with miR-206. Relative TM4SF1 mRNA (**D**) and protein (**E**) expression levels in HCT116. ^*^P<0.05, ^**^P<0.01, and ^***^P<0.001 compared with the miR-NC. The data are presented as mean ± standard error.

To investigate the mechanism of miR-206 targetting TM4SF1 in CRC cells, we determined that the TM4SF1 mRNA 3′-UTR contained miR-206-binding sites (Figure 3A). We constructed a luciferase reporter plasmid containing TM4SF1 3′-UTR with the conserved miR-206-binding site (TM4SF1 3′-UTR-WT) and a plasmid containing TM4SF1 3′-UTR with a miR-206 mutated target sequence (TM4SF1 3′-UTR-MT). The luciferase reporter assay results showed that co-transfected with miR-206 and the TM4SF1 3′-UTR-WT had significantly decreased luciferase activity compared with the NC group. However, the luciferase activity was not altered when miR-206 and TM4SF1 3′-UTR-MT were co-transfected into HCT116 and Caco-2 cells (Figure 3B). Then we detected the efficiency of miR-206 in HCT116 cells (Figure 3C). To investigate whether miR-206 regulates TM4SF1 expression, we transfected HCT116 cells with miR-206. The overexpression of miR-206 significantly suppressed TM4SF1 mRNA and protein expression by qRT-PCR and Western blot analysis, respectively (Figure 3D,E). These results suggest that TM4SF1 is a direct target of miR-206 in CRC cells.

### PGE2 regulates miR-206 and TM4SF1 mRNA expression in CRC cells

**Figure 4:**
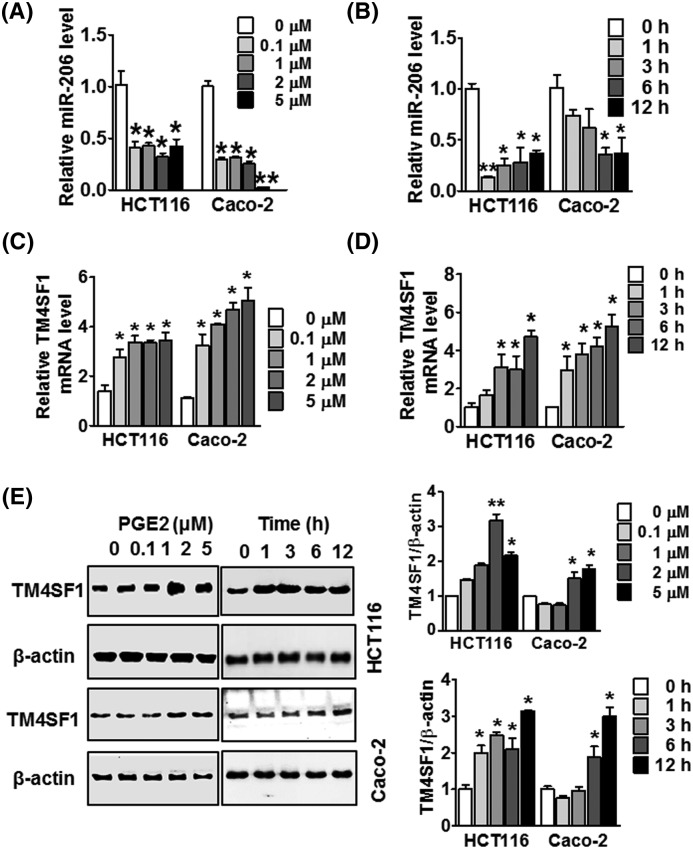
Effect of PGE2 at different concentrations and time points on miR-206 and TM4SF1 expression (**A**) HCT116 and Caco-2 cells were treated with the indicated concentrations of PGE2 (0, 0.1, 1, 2, and 5 μM, respectively) for 12 h, and miR-206 was detected by qRT-PCR. (**B**) miR-206 expression was detected by qRT-PCR, and HCT116 and Caco-2 cells were treated with PGE2 at a concentration of 1μM at the incubated time points. (**C**) HCT116 and Caco-2 cells were treated with the indicated concentrations of PGE2 (0, 0.1, 1, 2, and 5μM, respectively) for 12 h, and TM4SF1 expression was detected by qRT-PCR. (**D**) The expression of TM4SF1 was detected by qRT-PCR. HCT116 and Caco-2 cells were treated with PGE2 at a concentration of 1 μM at the indicated time points. (**E**) Expression of TM4SF1 protein was detected by Western blot analysis in HCT116 and Caco-2 cells in a time- and dose-dependent manner. ^*^P<0.05 and ^**^P<0.01 were compared with the control. The data are presented as mean ± standard error.

To identify whether PGE2 mediates the expression of miR-206 in CRC cells, we assessed the expression of miR-206 in HCT116 and Caco-2 cells with different concentrations of PGE2. As shown in Figure 4A, treatment with PGE2 suppressed the expression of miR-206 when incubated with PGE2 concentrations ranging from 0.1 to 5 μM as compared with the untreated group. PGE2 also suppressed the expression of miR-206 at the various time points in HCT116 and Caco-2 cells (Figure 4B). Next, to investigate whether PGE2 altered TM4SF1 expression, we treated these cells with PGE2 at different concentrations and time points. PGE2 dramatically enhanced expression of TM4SF1 mRNA and protein in a time- and dose-dependent manner compared with the untreated control (Figure 4C–E). These results suggest that PGE2 alters expression of miR-206 and TM4SF1 in HCT116 and Caco-2 cells.

### Effect of miR-206 on PGE2-induced cell proliferation

**Figure 5:**
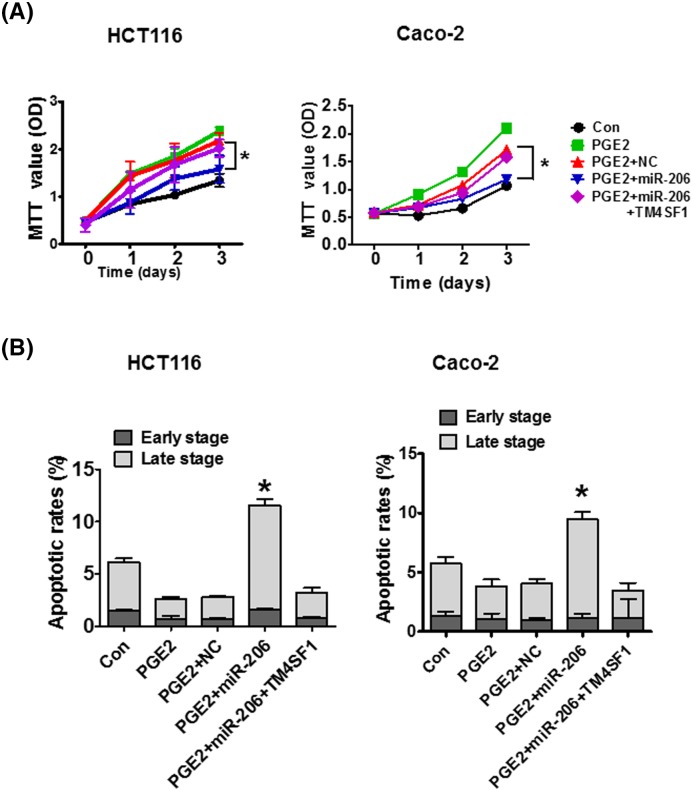
miR-206 suppresses CRC cell proliferation and induces cell apoptosis by targetting TM4SF1 in PGE2-induced cells (**A**) HCT116 and Caco-2 cells were transfected with miR-206 or TM4SF1 and treated with PGE2 for the indicated time. MTT assay was used to investigate the effect of miR-206 on the proliferation of PGE2-induced cells. (**B**) Apoptotic cells were determined using flow cytometry analysis. The data are presented as mean ± standard error. *P<0.05 compared with control.

To investigate the effect of miR-206 on PGE2-induced CRC cell proliferation, HCT116 and Caco-2 cells were transfected with miR-206 and treated with PGE2. MTT assay results revealed that PGE2 treatment significantly increased cell proliferation compared with untreated cells. However, the increased cell proliferation rate was reduced by miR-206 compared with the miR-NC. PGE2-induced HCT116 and Caco-2 cells co-transfected with TM4SF1 reversed these results (Figure 5A). We also measured the effect of miR-206 on cell apoptosis ability. When HCT116 and Caco-2 cells were treated with PGE2, the percentage of apoptotic cells decreased form 6.5 to 2.1% and 6.4 to 4.4%, compared with the control group, respectively. Overexpression of miR-206 significantly increased the apoptotic cell percentages from 2.6 to 11.8% and 4.0 to 9.3% compared with NC group in PGE2-induced cells. Co-transfection with TM4SF1 reversed the apoptotic rate in PGE2-induced HCT116 and Caco-2 cells (Figure 5B). These results demonstrate that PGE2 regulates CRC cell proliferation and apoptosis, and miR-206 may inhibit the proliferation and the induction of apoptosis by targetting TM4SF1 in PGE2-induced cells.

### Overexpression of miR-206 in PGE2-induced cells inhibits migration and invasion through TM4SF1

**Figure 6:**
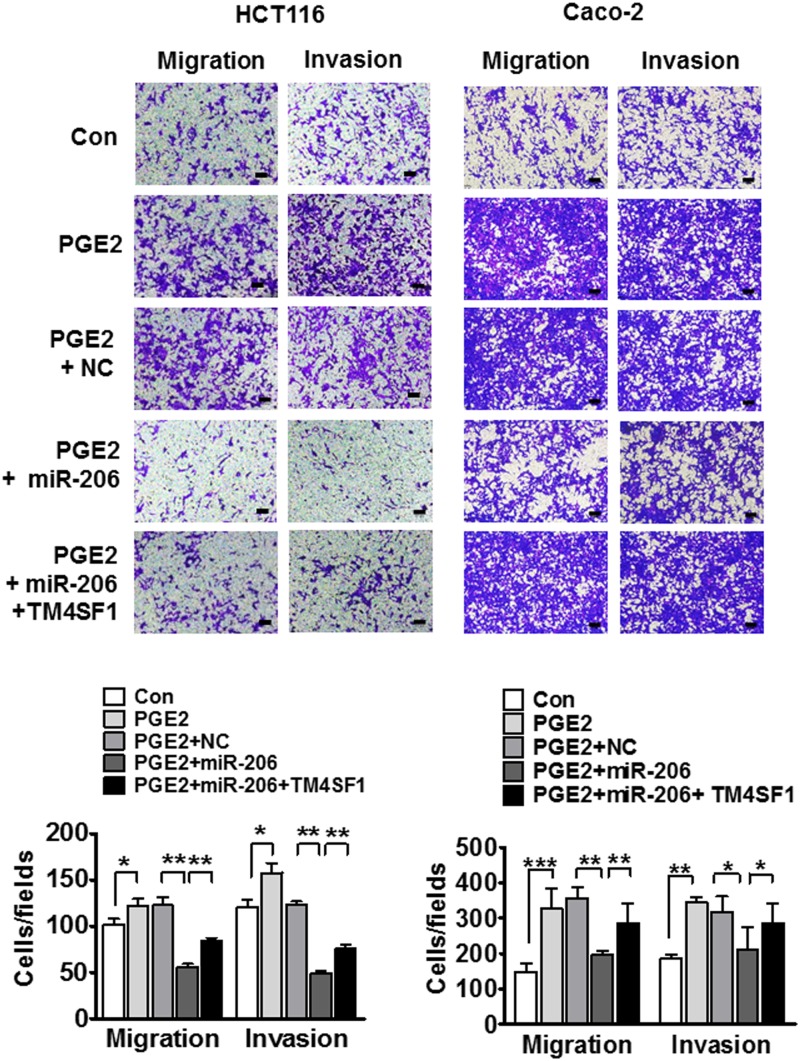
miR-206 inhibits cell migration and invasion in PGE2-induced cells Representative images show migrated cells (left) and invasive cells (right) and the number of moving tumor cells in HCT116 and Caco-2 cells. *P<0.05, **P<0.01, and ***P<0.001.

To further analyze whether miR-206 is involved in PGE2-induced EMT via targetting TM4SF1, we explored the migration and invasion properties of cells using a transwell migration and invasion assay. We found that PGE2 treatment significantly increased cell migration and invasion in HCT116 and Caco-2 cells. In addition, increased migration and invasion were significantly suppressed by miR-206 transfection. These results were also reversed when miR-206 and TM4SF1 were co-transfected into the PGE2-induced HCT116 and Caco-2 cells (Figure 6). Taken together, miR-206 targets TM4SF1 and suppresses migration and invasion in PGE2-induced HCT116 and Caco-2 cells.

### miR-206 inhibits TM4SF1 mRNA and protein expression in PGE2-induced cells

**Figure 7:**
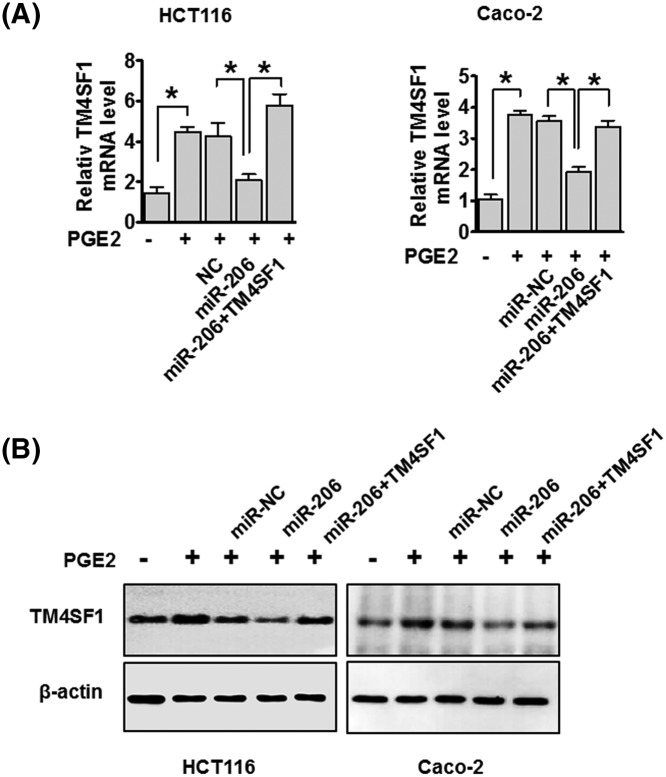
miR-206 suppresses TM4SF1 expression in PGE2-induced cells HCT116 and Caco-2 cells were transfected with either miR-206 or TM4SF1 plasmid in PGE2-induced cells. TM4SF1 mRNA and protein expression were detected by RT-PCR (**A**) and Western blot analysis (**B**). The data are presented as mean ± standard error. *P<0.05

To further determine whether miR-206 is involved in PGE2-induced EMT through targetting TM4SF1, we assayed TM4SF1 mRNA and protein expression using qRT-PCR and Western blot analysis, respectively. We found that PGE2 treatment increased the expression of TM4SF1 mRNA in HCT116 and Caco-2 cells. Subsequently, transfection of HCT116 and Caco-2 cells with miR-206 reduced TM4SF1 mRNA levels compared with the NC. Co-transfection with miR-206 and TM4SF1 recovered TM4SF1 mRNA expression (Figure 7A). Moreover, TM4SF1 protein expression in PGE2-induced CRC cells was suppressed by miR-206, and protein expression was recovered with miR-206 and TM4SF1 co-transfection (Figure 7B). These results suggest that miR-206 regulates both the transcriptional and translational levels of TM4SF1 in PGE2-induced CRC cells.

### Overexpression of miR-206 inhibits PGE2-induced EMT by down-regulation of TM4SF1 in CRC cells

**Figure 8:**
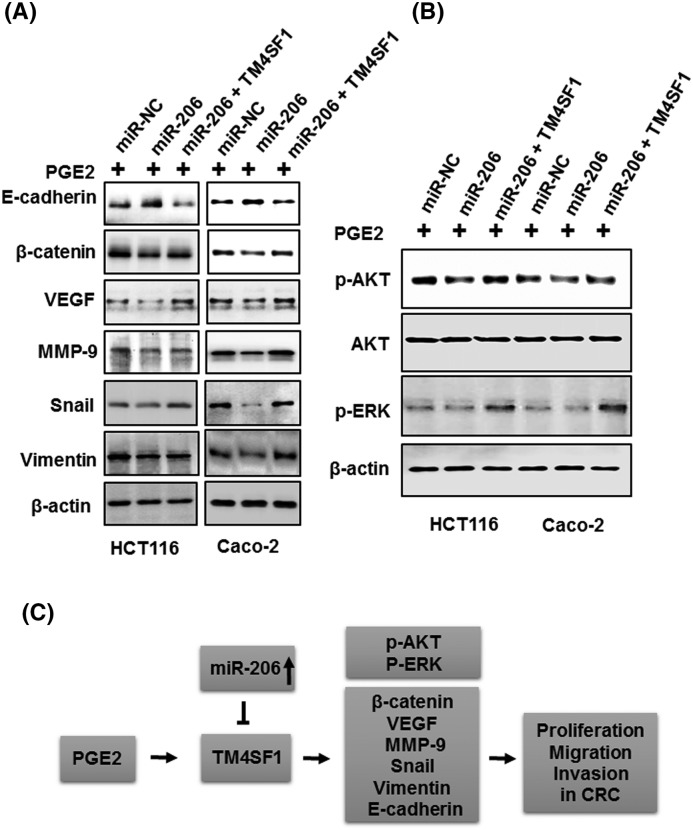
The effect of miR-206 overexpression can be reversed by the induction of TM4SF1 in PGE2-induced cells (**A**) HCT116 and Caco-2 cells were transfected with miR-206 or TM4SF1 and treated with PGE2 for 12 h. E-cadherin, β-catenin, VEGF, MMP-9, Snail, and Vimentin were detected. (**B**) The expression of *p*-AKT, AKT, *p*-ERK, PCNA, an β-actin loading control using Western blot analysis. (**C**) Schematic illustration of the proposed mechanism of miR-206-mediated inhibition of TM4SF1 in PGE2-induced colon cancer cells.

To further explore how miR-206 regulates PGE2-induced EMT, we determined the expression levels of EMT regulators E-cadherin, β-catenin, VEGF, MMP-9, Snail, and Vimentin. The increased protein levels were confirmed by treating PGE2 in HCT116 cells (not shown). As shown Figure 8A, the overexpression of miR-206 suppressed expression of β-catenin, VEGF, MMP-9, Snail, and Vimentin and enhanced E-cadherin expression compared with NC. Furthermore, the inhibitory effects of miR-206 were recovered by the overexpression of TM4SF1 in PGE2-induced HCT116 and Caco-2 cells. To further extend our analysis, we examined PGE2-stimulated signaling pathways, including MARK/ERK and PI3K/AKT. The overexpression of miR-206 reduced the phosphorylation levels of AKT and ERK, which were restored by transfection with TM4SF1 in PGE2-induced cells. Taken together, these results suggest that miR-206 may function as a negative regulator of cell proliferation, migration, and invasion in PGE2-induced CRC cells through the suppression of TM4SF1.

## Discussion

Cancer metastasis is a complex and multi-step process relying on tumor–stroma interactions [40]. In the present study, we investigated the role and molecular mechanism of TM4SF1 in PGE2-induced colon carcinogenesis.

PGE2 is an important prostaglandin that had been implicated in colorectal carcinogenesis [41]. PGE2 levels are commonly at higher concentrations in colon cancer tissues than in normal tissues [42]. Several studies have previously shown that EP receptor activation by PGE is important for cell migration in a number of cancer cells [11]. To determine the downstream receptor of PGE2, we analyzed mRNA expression of the PGE2 receptors, EP1, EP2, EP3, and EP4 in PGE2-stimulated HCT116 cells. We found that the expression levels of EP2 and EP4 receptors mRNAs were significantly increased in PGE2-induced cells compared with the untreated groups, which resulted in the up-regulation of TM4SF1 in CRC cells (not shown). Next, we analyzed the expression of COX-2 and the concentration of PGE2 in CRC tissues and serum, respectively. The expression of COX-2 was significantly increased in CRC tissues compared with adjacent normal tissues. Interestingly, there are increased serum levels of PGE2 in CRC patients compared with healthy controls. These results suggest that PGE2 may act as an important factor in the development and progression of CRC.

TM4SF1 is a plasma membrane and is enriched with TM4SF1 microdomains (TMED). Recently, there have been numerous studies of TM4SF1 in several malignant cancers, including liver, breast, and CRCs [38,43,44]. Our previous studies had found that TM4SF1 is highly expressed in human CRC tissues and various CRC cell lines. The expression of TM4SF1 is also associated with CRC migration and invasion *in vitro* [32,33]. Silencing of TM4SF1 showed increased apoptosis and reduced cell migration in human liver cancer cells and the overexpression of TM4SF1 increased tumor growth and metastasis *in vivo* [38]. Knockdown of TM4SF1 had decreased pancreatic tumor growth and increased responsiveness to treatments with gemcitabine in orthotopic pancreatic tumor models [40]. In the present study, we found that the expression of TM4SF1 mRNA and protein was up-regulated by treatment with PGE2. Moreover, the treatment of PGE2 significantly enhanced cell proliferation, migration, and invasion *in vitro*. These results suggest that TM4SF1 may have an essential role in the tumorigenesis and progression in PGE2-induced CRC cells. Furthermore, we discovered that miR-206 directly regulates TM4SF1, which functioned as a metastasis suppressor of miRNA in various cancers, including breast, lung, glioblastoma, and colon cancers, by binding the predicted seed sites [17,21,29,45]. In particular, the overexpression of miR-206 decreased mRNA and protein expressions of TM4SF1. Moreover, miR-206 expression was significantly down-regulated in CRC tissues and cell lines and was associated with lymph node metastasis. These results demonstrated that miR-206 may play a crucial role in the development of CRC by directly targetting TM4SF1.

Many reports have demonstrated that PGE2 changes tumor cell morphology and regulates tumor survival by inhibiting apoptosis and promoting bath cell proliferation and metastatic ability to increase tumor progression [11–14]. Our data showed that treatment of CRC cells with PGE2 significantly increased cell proliferation, migration, and invasion. Furthermore, increased proliferation, migration, and invasion were suppressed by the overexpression of miR-206 in PGE2-induced cells. However, co-transfecting PGE2-induced HCT116 and Caco-2 cells with miR-206 and TM4SF1 reversed these results. Consequently, we confirmed expressions of E-cadherin, β-catenin, VEGF, MMP-9, Snail, and Vimentin, which are essential regulators of EMT [38,46]. We found that treatment of CRC cells with PGE2 up-regulated the expression of β-catenin, VEGF, MMP-9, Snail, and Vimentin and down-regulated E-cadherin expression. In addition, increased expression was abrogated by transfection of PGE2 induced with miR-206, which also resulted in increased E-cadherin expression. Whereas, co-transfection of PGE2-induced CRC cells with miR-206 and TM4SF1 reversed these results.

PGE2 induction increased intracellular activation of ERK and PI3K/AKT signaling pathways, and regulated PGE2-dependent cell migration and survival [47,48]. Moreover, silencing of TM4SF1 suppressed breast cancer cell migration and invasion and induced apoptosis via inhibition of *p*-AKT/*p*-mTOR pathway [43]. miR-520f significantly down-regulated in HCC tissues and miR-520f/TM4SF1 axis-mediated cell proliferation and invasion through regulating PI3K/AKT [49].

Our results showed that expression of *p*-ERK and *p*-AKT in PGE2-induced cells is suppressed by overexpression of miR-206 and reversed by the overexpression of TM4SF1. Furthermore, we validated that miR-206 suppresses PGE2-induced cell proliferation, migration, and EMT process through *p*-AKT and *p*-ERK using MAPK/MAP kinase inhibitor (MEK, PD98059) or PI3K/AKT inhibitor (LY294002) in HCT116 cells (Supplementary Figure S2). These results suggested that miR-206/TM4SF1 may be associated with PGE2-induced cell proliferation, migration, and invasion through several molecules of EMT and signaling pathway, including E-cadherin, β-catenin, VEGF, MMP-9, Snail, Vimentin, *p*-AKT, and *p*-ERK.

The expression of miR-206 was inversely associated with TM4SF1 mRNA expression in CRC specimens, but the association was not significant and large-scale analysis is needed. We also need to examine the involvement of other signaling pathways and to conduct additional *in vivo* experiments.

In summary, our findings indicate that when CRC cells were stimulated with PGE2, TM4SF1 promoted cell proliferation, migration, and invasion. Through the binding of the TM4SF1 3′-UTR, miR-206 inhibited TM4SF1 expression and suppressed cell proliferation, migration, and invasion in PGE2-induced cells. Furthermore, we showed that EMT factors β-catenin, VEGF, MMP-9, Snail, and Vimentin were suppressed and increased E-cadherin by miR-206 in PGE2-induced CRC cells. miR-206 also suppressed *p*-ERK and *p*-AKT signaling pathways in PGE2-induced cells (Figure 8C). Taken together, these results suggest that miR-206/TM4SF1 may be a potential therapeutic target in PGE2-induced CRC cells.

## Supporting information

**Supplementary figure 1.** TM4SF1 promotes proliferation and migration of colon cancer cells.

**Supplementary figure 2.** miR-206 decreases PGE2-induced cell proliferation, migration, and EMT process through p-AKT and p-ERK pathway on colon cancer cells.

## Abbreviations

ATCC: American Type Culture Collection
β2M: β-2 microglobulin
COX-2: cyclooxygenase 2
CRC: colorectal cancer
c-MET: c-mesenchymal epithelial transition factor
DMEM: Dulbecco modified Eagle’s media
EMT: epithelial–mesenchymal transition
EMT-TF: EMT transcription factor
EP: prostaglandin E2 receptor
ERK: extracellular signal regulated kinases
HDAC6: histone deacetylases 6
HGF: hepatocyte growth factor
HNSCC: head and neck squamous cell carcinoma
JAK2/STAT3: janus kinase/signal transducer and activator of transcription 3
KLF4: Kruppel-like factor 4
MAPK: mitogen activated protein kinase
miR-206: micro RNA-206
MMP-9: matrix metalloproteinase-9
mPGES-1: microsomal prostaglandin E synthase-1
mTOR: mammalian target of rapamycin
NP-40: nonyl phenoxypolyethoxylethanol
OD: optical density
PGE2: prostaglandin E2
PCNA: proliferating cell nuclear antigen
PI3K: phosphoinositide 3-kinase
PTEN: phosphatase and tensin homolog
qRT-PCR: quantitative polymerase chain reaction
SD: standard deviation
TGF-β: tranforming growth factor beta
TM4SF1: transmembrane 4 L six family member 1
TMED: TM4SF1 microdomain
UTR: untranslated region
VEGF: vascular endothelial growth factor
WT: wild type
