# Supplementary material for: MiRNA-206 suppresses PGE2-induced colorectal cancer cell proliferation, migration, and invasion by targetting TM4SF1
Source: Biosci Rep. 2018 Sep 19;38(5):BSR20180664. doi: 10.1042/BSR20180664 (PMC6146292; doi:10.1042/BSR20180664)
Supplement: Supplementary file 1 [file bsr20180664_Supp1.pdf]

Supplementary figure 1

A

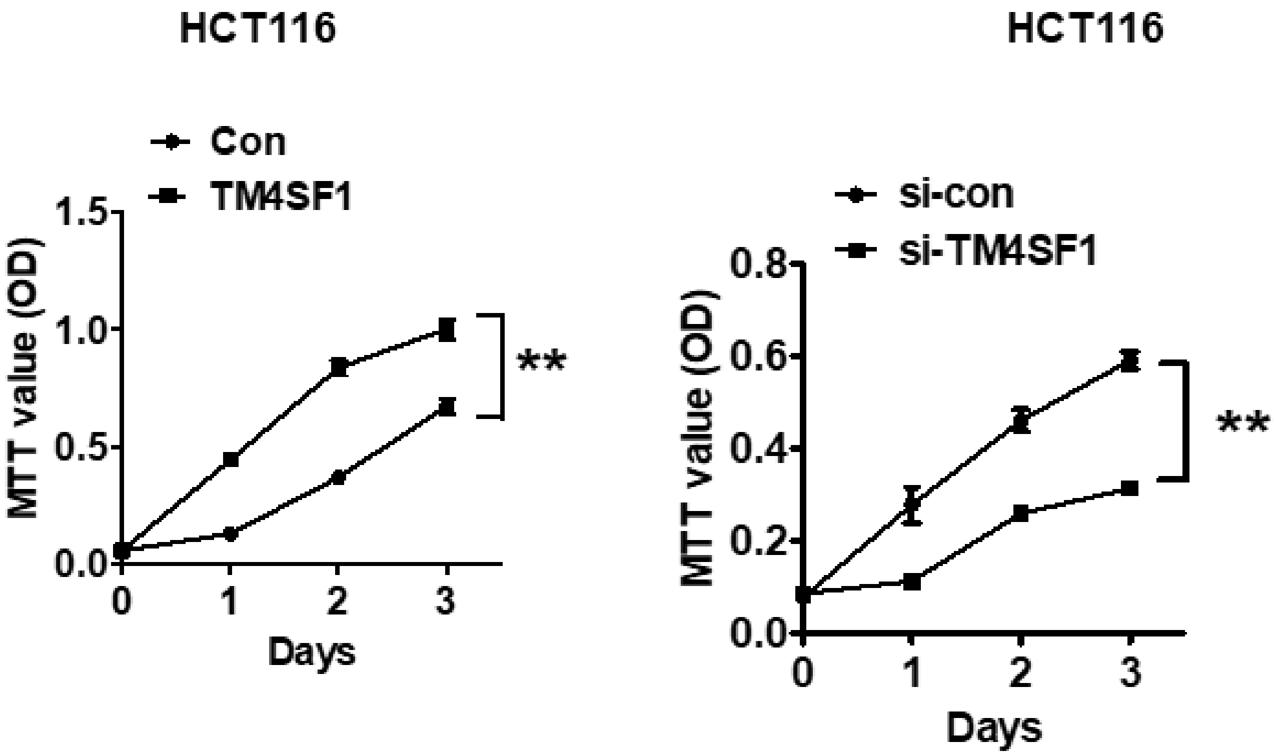

B

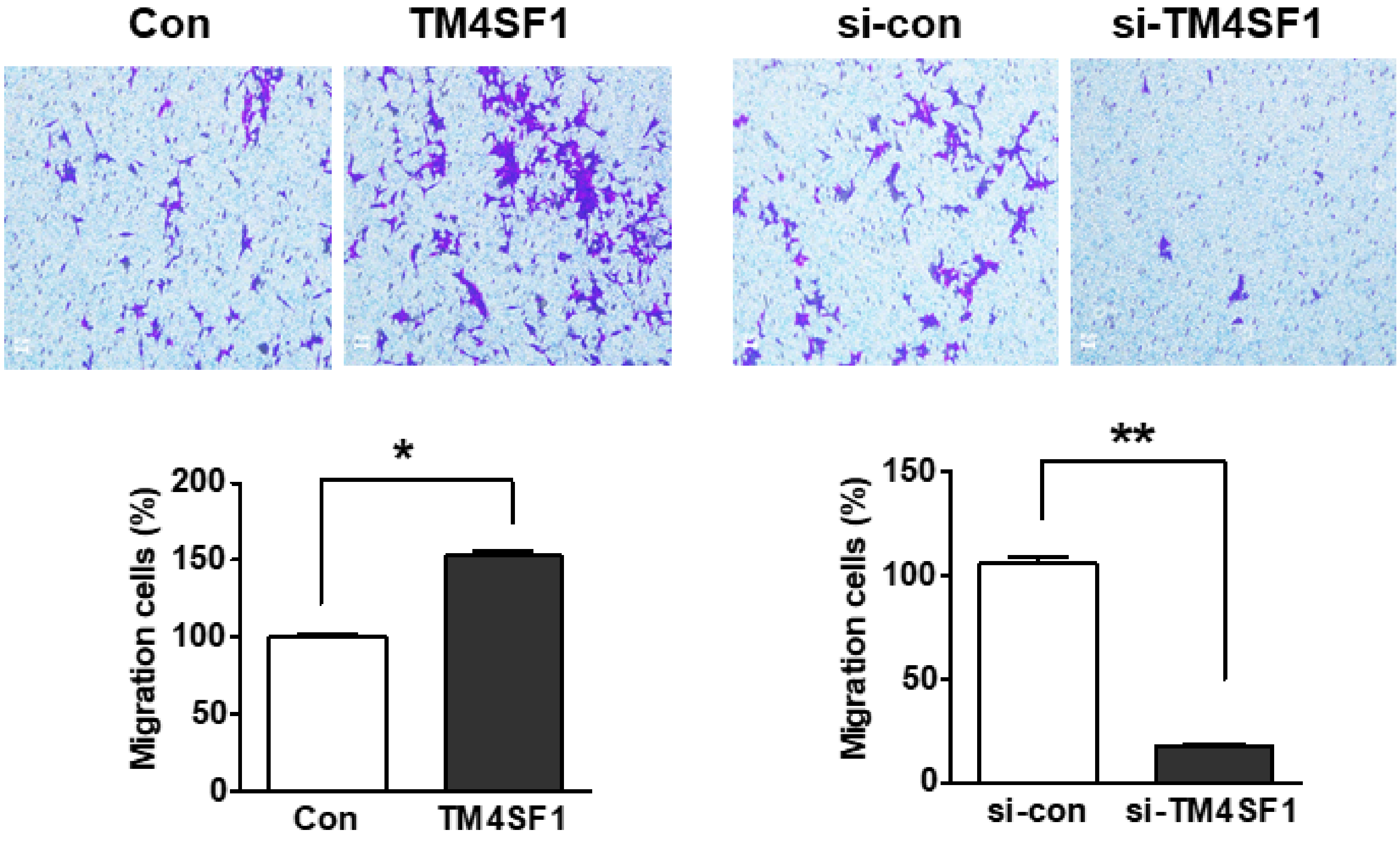

Supplementary figure 1

C

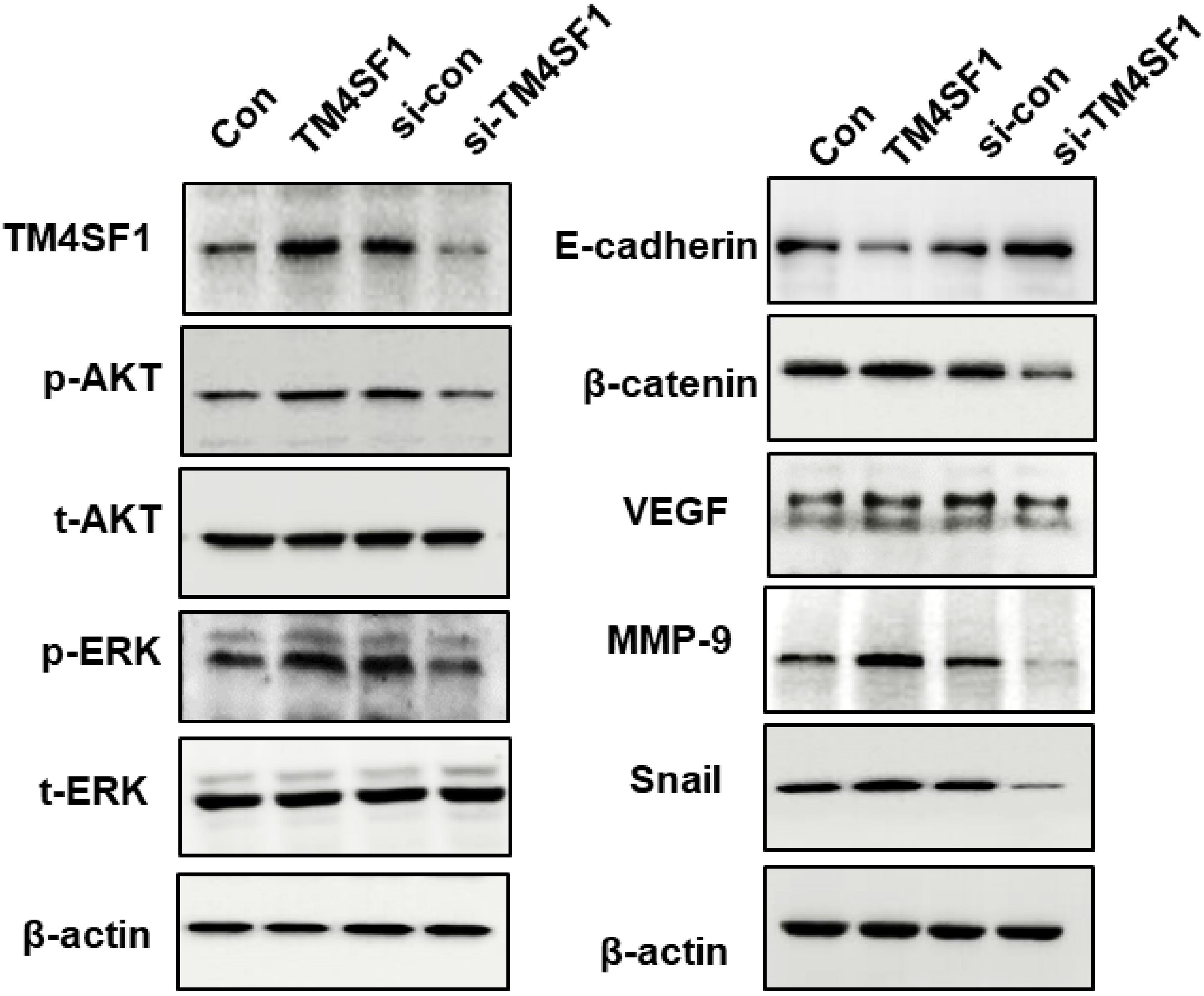

Supplementary figure 2

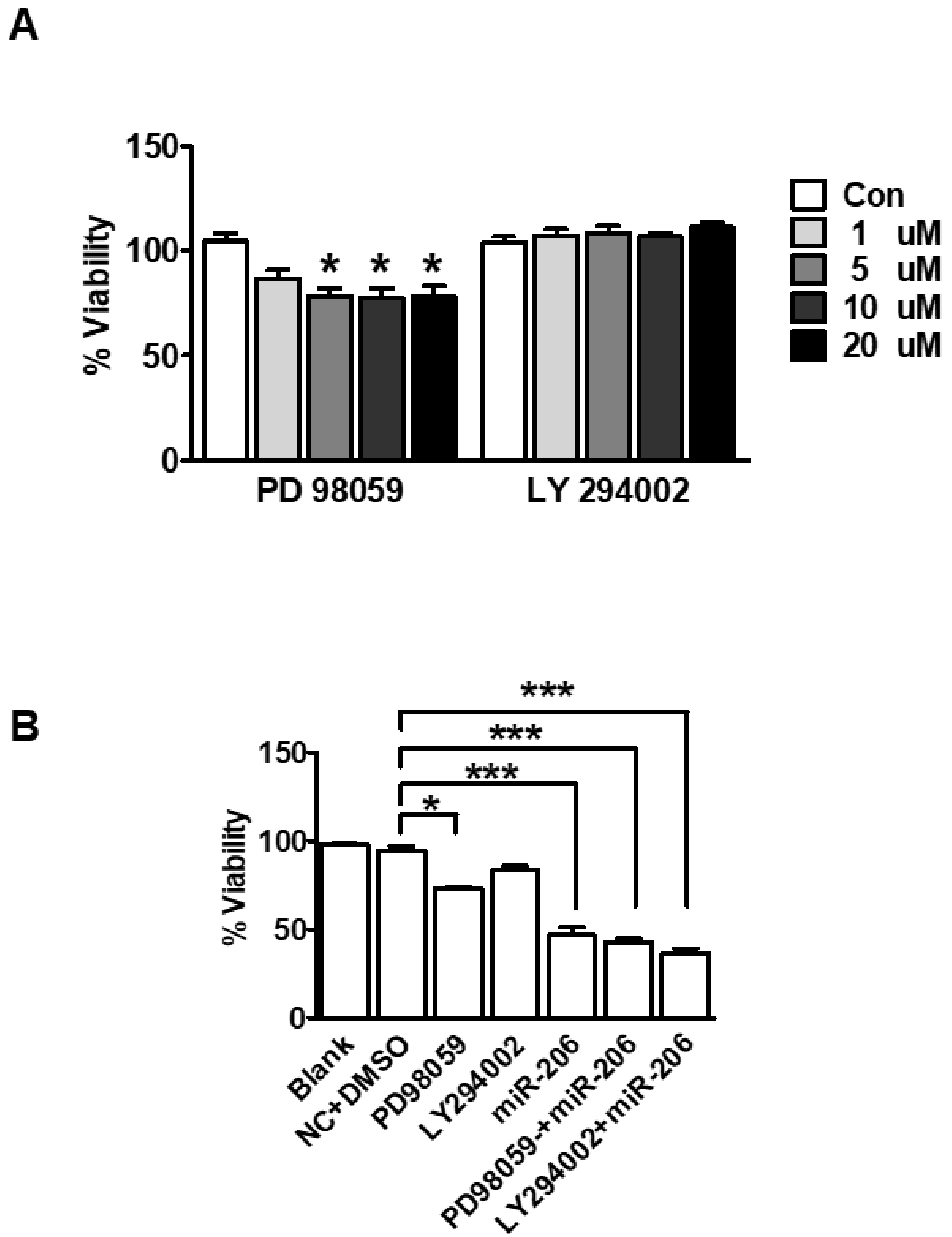

Supplementary figure 2

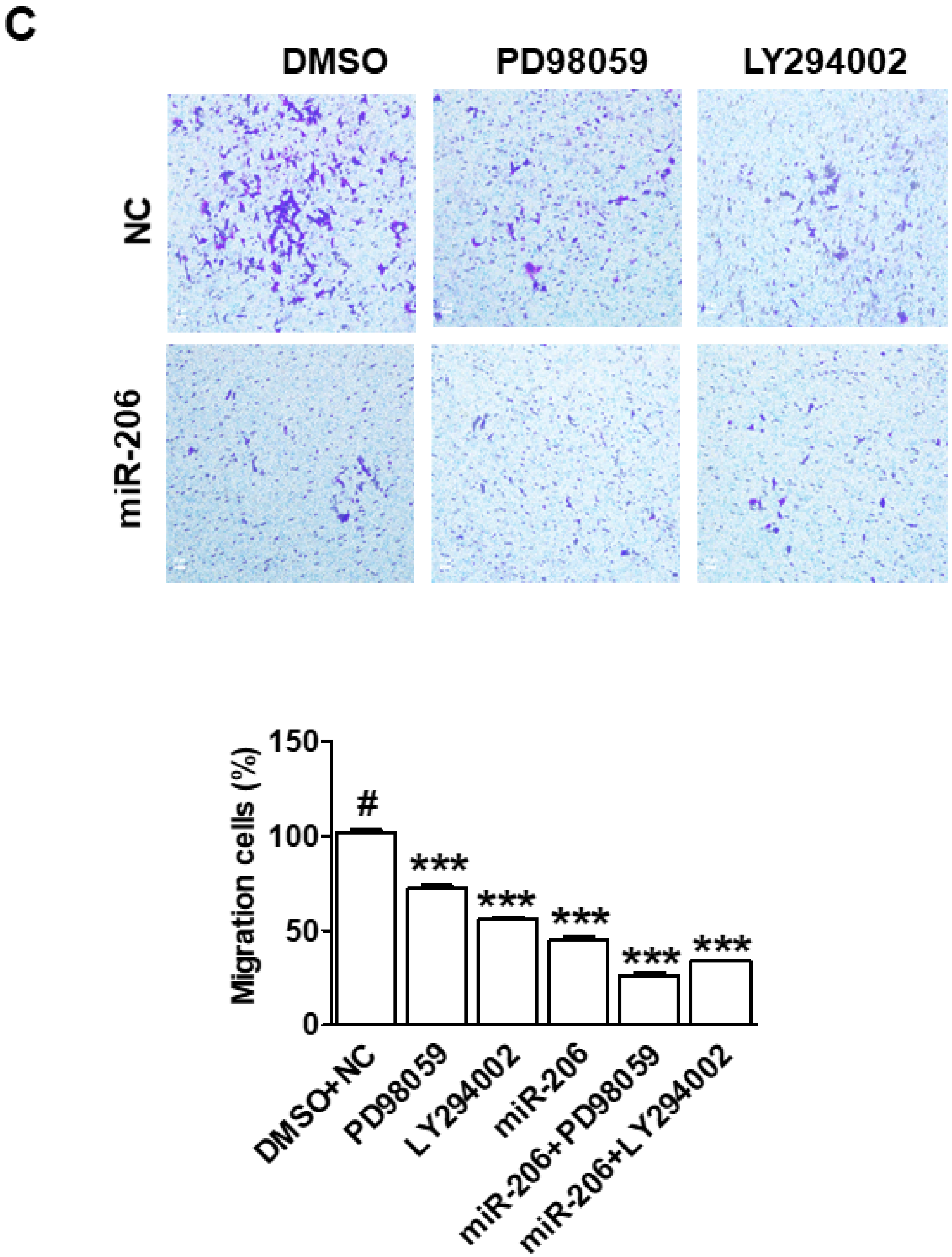

Supplementary figure 2

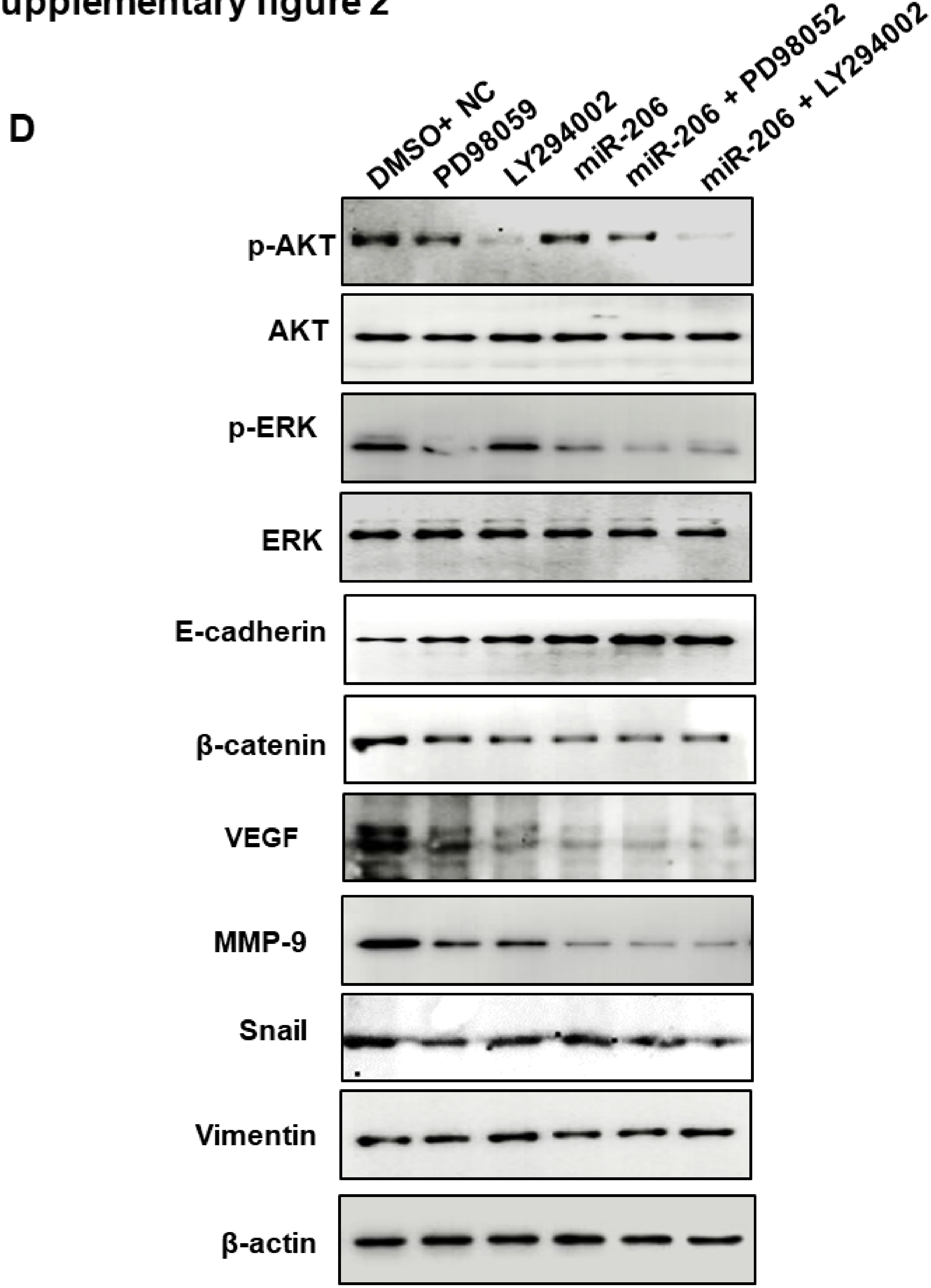

Supplementary figure 1.

TM4SF1 promotes proliferation and migration of colon cancer cells.

(A) HCT116 cells were transfected with TM4SF1 plasmid or si-TM4SF1 for 0, 1, 2, 3 days. The cell proliferation was determined using MTT assay. (B) The transwell migration assay was performed on HCT116 cells transfected with TM4SF1 plasmid or si-TM4SF1. (C) Western blot analysis was used to measure the protein levels of EMT markers or p-AKT and p-ERK signaling pathway in HCT116 cells. β-actin was detected as a loading control. * $p<0.05$, ** $p<0.01$.

Supplementary figure 2.

miR-206 decreases PGE2-induced cell proliferation, migration, and EMT process through p-AKT and p-ERK pathway on colon cancer cells.

(A) HCT116 cells were treated with PD98059 (MEK inhibitor) or LY294002 (PI3K inhibitor) under various concentration (0, 1, 5, 10, 20 μM) for 24 h. The cell viability was determined by MTT assay. * $p<0.05$ compared with con (0 μM). HCT116 cells were transfected with miR-206 for 48 h and treated with PD98059 (5 μM) or LY294002 (10 μM) for 24 h, and then PGE2 was treated for 12 h, and then determined using MTT assay (B) and transwell migration assay (C). (D) The protein levels of p-AKT, p-ERK, E-cadherin, β-catenin, VEGF, MMP-9, Snail and Vimentin in HCT116 cells were detected by performing immunoblotting. The data are presented as mean ± S. D. of three independent experiments.
